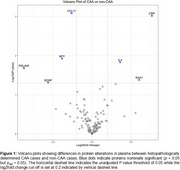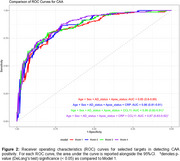# Blood‐based biomarker for identifying post‐mortem confirmed Cerebral Amyloid Angiopathy

**DOI:** 10.1002/alz70856_105227

**Published:** 2026-01-09

**Authors:** Alpana Singh, Marisa N. Denkinger, Kari Dieckhoff, James Liu, Geidy E Serrano, Thomas G Beach, Antoine Leuzy, Alirez Atri, Eric M. Reiman, Nicholas J. Ashton

**Affiliations:** ^1^ Banner Sun Health Research Institute, Sun City, AZ, USA; ^2^ Banner Alzheimer's Institute, Phoenix, AZ, USA

## Abstract

**Background:**

Cerebral amyloid angiopathy (CAA) is a cerebrovascular disorder characterized by the deposition of amyloid‐β (Aβ) in the walls of leptomeningeal and cortical blood vessels leading to increased risk of microbleeds, intracerebral hemorrhages, and progressive cognitive decline. It is estimated that up to 90% of individuals with Alzheimer's disease (AD) exhibit some degree of CAA. Notably, CAA has been identified as a major contributor to the risk of Amyloid‐Related Imaging Abnormalities (ARIA), particularly in patients undergoing treatment with anti‐amyloid therapies. The use of blood‐based biomarkers to accurately detect and assess the severity of CAA is crucial for tailoring treatment plans while reducing the risk of adverse effects.

**Method:**

We employed the Nucleic Acid‐Linked Immuno‐Sandwich Assay (NULISA™) central nervous system panel for an exploratory biomarker quantification in plasma of patients with CAA or no‐CAA (presence of cerebral amyloidotic blood vessels) utilizing samples from the Banner Health Brain and Body Donation Program (BBDP) We evaluated the differential protein expression between groups using a linear model. This model was adjusted for age, sex, *APOE* e4 carrier status and the presence of Alzheimer's amyloid plaque load (AD‐status).

**Result:**

We selected 251 participants (age: 85±8.2) from the BBDP cohort with a plasma sample taken < 5 years (1.45±1.26) prior to death. At post‐mortem each case was classified as CAA+ (*n* = 140) or CAA‐ (*n* = 111). NULISA™ identified several novel proteomic biomarkers which were up‐regulated (CRP, IL4, SAA1), and down‐regulated (CCL11, PDLIM5, NPY and GDNF) in CAA pathology carriers (Figure 1). We further performed receiver operating characteristic (ROC) curve analysis comparing models and found that a model including age, sex, AD‐status, APOE e4 status, CRP and CCL11 had an area under the curve (AUC) of 0.87 (95%CI, 0.83‐0.92) to identify the presence of CAA at post‐mortem (Figure 2).

**Conclusion:**

Blood‐based biomarkers capable of identifying CAA could play an important role in improving treatment outcomes by highlighting ARIA risk prior to treatment initiation. We found that plasma proteins related to inflammation, blood brain barrier dysfunction and cytoskeletal stability were significantly changed in participants with confirmed CAA. Further work will be required to replicate these findings in an independent dataset.